# Molecular Targets in *Streptococcus pyogenes* for the Development of Anti-Virulence Agents

**DOI:** 10.3390/genes15091166

**Published:** 2024-09-04

**Authors:** Kyu Hong Cho

**Affiliations:** Department of Biology, Indiana State University, 600 Chestnut St. S224, Terre Haute, IN 47809, USA; kyuhong.cho@indstate.edu; Tel.: +1-(812)-237-2412

**Keywords:** *Streptococcus pyogenes*, antibiotic resistance, RopB, Mga, CovRS, CdaA, Pde2, c-di-AMP

## Abstract

*Streptococcus pyogenes*, commonly known as Group A Streptococcus (GAS), is a significant human pathogen responsible for a wide range of diseases, from mild pharyngitis to severe conditions such as necrotizing fasciitis and toxic shock syndrome. The increasing antibiotic resistance, especially against macrolide antibiotics, poses a challenge to the effective treatment of these infections. This paper reviews the current state and mechanisms of antibiotic resistance in *S. pyogenes*. Furthermore, molecular targets for developing anti-virulence agents, which aim to attenuate virulence rather than killing it outright, are explored. This review specifically focuses on virulence regulators, proteins that coordinate the expression of multiple virulence factors in response to environmental signals, playing a crucial role in the pathogen’s ability to cause disease. Key regulatory systems, such as RopB, Mga, CovRS, and the c-di-AMP signaling system, are discussed for their roles in modulating virulence gene expression. Additionally, potential molecular target sites for the development of anti-virulence agents are suggested. By concentrating on these regulatory pathways, it is proposed that anti-virulence strategies could enhance the effectiveness of existing antibiotics and reduce the selective pressure that drives the development of resistance.

## 1. Introduction

Antibiotic resistance is a major global health concern, arising when bacteria develop mechanisms to withstand drugs intended to kill them or inhibit their growth. This resistance compromises the effectiveness of antibiotics, which are crucial in treating bacterial infections, preventing surgical sepsis, and enabling chemotherapy. *S. pyogenes*, also known as Group A Streptococcus (GAS), is a significant pathogen responsible for diseases ranging from mild pharyngitis (strep throat) to severe invasive diseases like necrotizing fasciitis and toxic shock syndrome. Currently, there are no commercial vaccines available for *S. pyogenes* infections, and treatment relies heavily on antibiotics. However, like other pathogens, *S. pyogenes* has developed resistance to several antibiotics, including macrolides such as erythromycin and azithromycin, tetracyclines, and fluoroquinolones. Although *S. pyogenes* remains susceptible to penicillin, some concerns persist. Approximately 10% of the U.S. population reports a penicillin allergy, with higher rates observed among older adults and hospitalized patients [1]. Clinically, around 30% of pharyngitis cases are not fully cured with antibiotic treatment, resulting in recurrent infections [2,3]. Additionally, some strains have shown increasing resistance to penicillin at subclinical doses. Therefore, the development of new preventive or therapeutic measures effective against *S. pyogenes* infections is crucial. Anti-virulence drugs, which target bacterial virulence factors without killing the bacteria directly, are a promising avenue for developing new treatments against *S. pyogenes* infections.

Anti-virulence agents represent a promising strategy in combating bacterial infections by targeting bacterial virulence factors rather than growth pathways. These agents disarm bacteria, rendering them less harmful without killing them directly. By preventing bacterial adhesion to host tissues, interfering with bacterial toxins, and modulating the expression of virulence genes, anti-virulence agents can significantly attenuate bacterial pathogenicity [4]. One of the key benefits of anti-virulence strategies is their potential to reduce the likelihood of resistance development. Unlike traditional antibiotics, which exert lethal pressure on bacteria, anti-virulence agents do not directly threaten bacterial survival, thereby reducing the evolutionary drive for resistance [5]. Additionally, these agents can be highly specific, targeting unique bacterial virulence pathways without affecting the host’s beneficial microbiota [5]. Anti-virulence strategies may also resensitize bacteria to antibiotics against which they have previously developed resistance. For instance, *Pseudomonas aeruginosa* utilizes quorum sensing to regulate biofilm formation and virulence factor production. Quorum sensing inhibitors (QSIs) can disrupt these processes, leading to increased susceptibility of *P. aeruginosa* to antibiotics like tobramycin, which is often less effective against biofilm-associated infections [6]. In mild infections, anti-virulence agents might be sufficient to clear the infection with the aid of host immunity. However, these agents alone may not be enough to clear severe or acute infections requiring rapid bacterial killing. In such cases, they can be combined with antibiotics, working synergistically to enhance antibiotic effectiveness and potentially lower the required antibiotic doses [4].

In this review, the current state of antibiotic resistance development in *S. pyogenes* is briefly described first. Then the molecular targets for developing anti-virulence drugs aimed at treating *S. pyogenes* infections are discussed.

## 2. The Development of Antibiotic Resistance by *S. pyogenes*

### 2.1. Resistance to Macrolides

Penicillin is the first-choice antibiotic for treating *S. pyogenes* infections; however, other antibiotics are used under specific conditions, such as for patients allergic to penicillin, where erythromycin and other macrolides are recommended as alternative treatments.

Macrolide resistance in *S. pyogenes* is often mediated by the acquisition of *erm* (erythromycin ribosome methylase) genes, whose products modify the ribosomal target of the antibiotic, preventing its action by methylating the 23S rRNA component of the 50S ribosomal subunit [7]. This modification inhibits erythromycin from effectively binding to the ribosome, thereby blocking protein synthesis. The most common *erm* genes in *S. pyogenes* include *ermB*, *ermT*, and *ermTR* (an *ermA* subclass) [8]. These genes confer resistance not only to erythromycin but also to other macrolides, lincosamides, and streptogramin B antibiotics, known as MLS_B_ resistance [9]. The *erm* genes spread through mechanisms such as transposons (in the case of most *ermB*), integrative and mobilizable elements (*ermTR*), or plasmids (*ermT*) [9].

Another mechanism of macrolide resistance involves the efflux of erythromycin out of the bacterial cell, reducing its intracellular concentration to sub-inhibitory levels. This is facilitated by efflux pump systems encoded by *mef* (macrolide efflux) genes, leading to multidrug resistance by expelling various antibiotics [8]. The most prevalent *mef* gene in *S. pyogenes* is *mefA*. The *mefA* gene is linked to the M phenotype, characterized by resistance to 14- and 15-membered macrolides (like erythromycin and azithromycin) while remaining susceptible to 16-membered macrolides, lincosamides, and streptogramin B antibiotics [10]. The *mefA* gene in *S. pyogenes* is frequently associated with prophages [10,11].

### 2.2. Resistance to Tetracycline

Tetracycline resistance in *S. pyogenes*, like in many other bacteria, primarily involves the acquisition and expression of tetracycline resistance genes. These genes encode proteins that protect the bacterial ribosome from tetracycline inhibition or actively pump the antibiotic out of the cell.

The most common mechanism of tetracycline resistance in *S. pyogenes* involves the acquisition of *tetM* and *tetO* genes. These genes encode ribosomal protection proteins that prevent tetracycline from binding to the ribosome, thereby allowing protein synthesis to continue in the presence of the antibiotic [12]. TetM and TetO proteins are similar to elongation factors EF-Tu and EF-G and work by binding to the ribosome and inducing a conformational change that displaces tetracycline from its binding site. This action allows translation to proceed even in the presence of tetracycline [13].

Another mechanism involves efflux pumps, encoded by genes such as *tetK* and *tetL*, which actively transport tetracycline out of the cell. However, this mechanism is less common in *S. pyogenes* compared to the ribosomal protection mechanism [14]. These efflux pumps are membrane-associated proteins that use the proton motive force to expel tetracycline from the bacterial cell, reducing the intracellular concentration of the antibiotic to sub-inhibitory levels.

Most *tet* resistance genes are located on mobile genetic elements, such as transposons, plasmids, or integrative and conjugative elements (ICEs), facilitating their horizontal transfer between bacteria. They are often co-located with *erm* or *mef* genes [15].

### 2.3. Resistance to Fluoroquinolone 

Fluoroquinolones, such as ciprofloxacin, may be considered an alternative therapy for *S. pyogenes* infections if the initial treatment with penicillin fails. Although rare, fluoroquinolone resistance has been reported in *S. pyogenes*. This resistance typically arises from mutations in the quinolone resistance-determining regions (QRDRs) of DNA gyrase and topoisomerase IV [16].

### 2.4. Increased Subclinical Resistance to Penicillin

Penicillin remains the drug of choice for treating *S. pyogenes* infections, as clinically administered doses are effective against all *S. pyogenes* strains. This is in contrast to other streptococcal pathogens, such as *Streptococcus pneumoniae*, where penicillin resistance due to mutations in penicillin-binding proteins (PBPs) is well documented. However, some *S. pyogenes* strains have developed resistance to subclinical concentrations of penicillin. These strains exhibit mutated PBPs and increased minimum inhibitory concentrations (MICs) of penicillin, indicating resistance rather than tolerance or persistence [17].

Mutations in genes encoding penicillin-binding proteins (PBPs), which are the target of β-lactam antibiotics, can lead to reduced affinity for these drugs. Penicillin-resistant *S. pneumoniae* strains usually exhibit mutations in several PBPs, commonly PBP1a, PBP2x, and PBP2b [18,19]. Initial resistance often involves mutations in PBP2x, followed by additional mutations in PBP2b and other PBPs for higher levels of resistance [20]. The cumulative effect of these mutations leads to significant resistance levels, as each altered PBP contributes to the overall reduction in antibiotic binding and efficacy. These changes are often the result of genetic recombination events where *S. pneumoniae* acquires altered PBP genes from other streptococcal species [21].

Several studies indicate that *S. pyogenes* strains with increased resistance to penicillin are emerging [22,23]. These strains contain missense mutations in the *pbp2x* gene, which is the first step in developing antibiotic resistance in other streptococci. It was previously believed that mutations leading to low-affinity penicillin-binding proteins (PBPs) would impose a fitness cost on *S. pyogenes*. One naturally occurred missense mutation, T553K in PBP2x, increases the MICs for ampicillin and amoxicillin eightfold (but not to the level of clinical resistance). However, this mutation did not impact bacterial growth in vitro [22]. Moreover, *S. pyogenes* mutants with P601L mutation in PBP2x that also confer increased resistance to β-lactams show enhanced growth in vitro [23]. These findings highlight the importance of vigilant monitoring for β-lactam resistance phenotypes in *S. pyogenes*.

Despite the widespread presence of penicillinase genes in other bacterial species, *S. pyogenes* has not acquired penicillinase genes. While horizontal gene transfer (HGT) is common among bacteria, certain species have barriers that limit the acquisition of foreign DNA. *S. pyogenes* may have genetic mechanisms that restrict the uptake and incorporation of penicillinase genes from other bacteria. The transfer of resistance genes often involves mobile genetic elements like plasmids, transposons, and integrative and conjugative elements (ICEs). *S. pyogenes* may lack the compatibility factors required for the stable acquisition and maintenance of penicillinase genes.

## 3. Molecular Targets for Developing Anti-Virulence Agents against *S. pyogenes* Infections

Developing new anti-virulence agents targeting *S. pyogenes* involves identifying molecular targets essential for its virulence. Targeting multiple virulence factors, rather than focusing on a single factor, would enhance the effectiveness of these agents. *S. pyogenes* possesses unique signaling pathways that control several virulence factors, offering an opportunity to target these pathways instead of individual virulence factors. Each virulence factor often shares common enzymatic functions with host proteins, such as proteases, DNases, RNases, and lipases, complicating the development of specific inhibitors. However, the signaling pathways in *S. pyogenes* that do not exist in human hosts minimize the risk of affecting host proteins. These pathways usually have a master regulator, and inhibiting the master regulator can significantly attenuate the virulence of *S. pyogenes*. By focusing on these unique signaling pathways and their master regulators, we might be able to develop highly specific anti-virulence agents that effectively neutralize the threat of *S. pyogenes*. This review focuses on master regulators of the signaling pathways that govern the pathogenicity of *S. pyogenes*.

To evaluate the effectiveness of anti-virulence agents, it is essential to use appropriate animal models. Since *S. pyogenes* can cause a range of diseases, from skin infections to systemic conditions, different models have been tailored to study specific aspects of these infections, enhancing our understanding of the pathogen’s behavior in various contexts (for an extensive review, see [24]). Murine models are primarily used to study skin infections and inflammatory responses, such as subcutaneous ulcers. These models are cost-effective and easily manipulated genetically. Non-human primate models are utilized to investigate pharyngitis and its progression to rheumatic fever. These primate models can develop type-specific humoral immune responses and exhibit significant pharyngeal symptoms, making them more relevant for studying human-like infections. However, these primate models are rarely employed due to their high cost and limited availability. Each model has distinct strengths and limitations, so researchers should select the appropriate model based on the specific disease aspect under investigation to ensure findings can be effectively extrapolated to human disease.

### 3.1. RopB

RopB (Regulator of Proteinase B), also known as Rgg, is a transcriptional regulator in *S. pyogenes* that plays a crucial role in the pathogenesis and regulatory networks. It integrates signals from the growth environment and metabolic state to control the expression of virulence factors, thereby influencing the pathogenicity of *S. pyogenes*. 

RopB modulates the expression of various virulence factors in response to different growth phases and environmental conditions. Specifically, RopB induces the expression of the SpeB protease during the stationary phase [25,26]. SpeB is a cysteine protease that degrades host tissues and aids in immune evasion. The inactivation of RopB in the NZ131 strain increases the transcription of the virulence operon, which includes the NAD-glycohydrolase (*spn*), immunity factor (*ifs*), and streptolysin O (*slo*) [27]. This inactivation also enhances the transcription of other virulence-associated genes, such as those encoding a superantigen (*speH*), adhesion protein (*scl1*/*sclA*), streptokinase (*ska*), capsule (*hasABC*), DNases (*mf and mf3*), and cell surface immune evasion factors (*grab* and *mac*). Additionally, it affects Mga regulon targets (*emm*, *scpA*, *sagA,* and *slo*) [28]. The expression of several key regulatory genes such as *mga*, *covRS*, and *fasBCAS* also changed in the *ropB* knockout strain, indicating that RopB influences these global regulators, thereby impacting the pathogenicity of *S. pyogenes* [28].

RopB senses environmental signals, particularly changes in pH, to control virulence factors in *S. pyogenes* through a mechanism involving a histidine switch. Specifically, the histidine residue at position 144 (H144) in RopB is critical for sensing environmental acidification. When the pH decreases, this histidine switch induces pH-dependent reorganizations of intramolecular interactions within RopB, which enhances its ability to bind to the SpeB-inducing peptide (SIP) [29]. The binding of SIP to RopB promotes oligomerization of RopB, which is essential for the activation of the SIP autoinduction circuit. This leads to the upregulation of *speB* expression. The interplay between the pH-sensing mechanism and the SIP signaling pathway allows the bacteria to effectively coordinate virulence factor production in response to environmental conditions, such as during high bacterial population density and acidic environments encountered in infected tissues.

**Figure 1:**
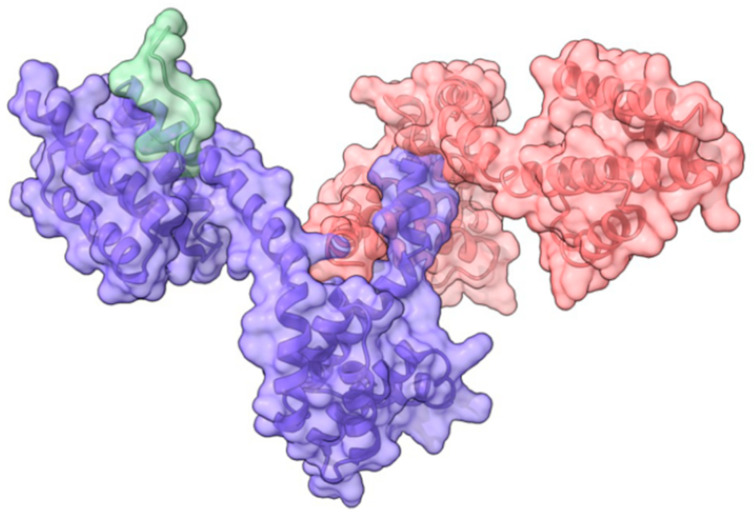
Structure of the dimer of RopB–CTD (C-terminal domain, amino acids 56–280) bound to the SpeB-Inducing Peptide (SIP). The structure is illustrated based on the crystal structure data of 6DQL available in the RCSB Protein Data Bank (RCSB PDB) and was generated using the Chimera X-1.8 software. RopB molecules are represented in purple and pink, while SIP is depicted in green. SIP is capable of binding to both RopB monomers; only one SIP bound to the purple RopB monomer is shown in this illustration.

There is considerable serotype variation in the RopB regulon, where different alleles affect the regulator’s function and, consequently, the expression of virulence genes [30]. This variability can lead to differences in virulence and disease manifestations among various *S. pyogenes* strains. Disrupting or inhibiting RopB function significantly decreases the overall virulence of the *S. pyogenes* NZ131 strain [29,31]. Given RopB’s central role in regulating virulence factors, it could be a potential target for therapeutic interventions aimed at mitigating the pathogenic effects of *S. pyogenes*. Inhibitors or modulators of RopB activity could potentially reduce virulence and improve treatment outcomes. One potential target site on RopB for developing inhibitors is the SIP binding site (Figure 1). If this site is occupied by a chemical agent, RopB may not be able to bind to SIP, potentially leading to a failure in activation. Another potential target site is the RopB dimerization domain. However, the roles of this domain in binding SIP, the pH-sensing histidine switch, or the oligomerization of RopB on the promoter, which are essential for the transcriptional activation by RopB, have not yet been studied.

### 3.2. Mga

Mga (multigene activator) is a transcriptional regulator that plays a crucial role in the pathogenicity of *S. pyogenes* by orchestrating the expression of numerous virulence factors. The Mga regulon is essential for the bacterium’s ability to colonize and infect host tissues. The genes in the regulon encode for surface proteins such as M protein, M-like surface proteins (Mrp and Enn), and fibronectin-binding adhesin (Fba), as well as secreted enzymes including the cysteine protease SpeB, C5a peptidase (ScpA), and the secreted inhibitor of complement (Sic) [32,33,34,35]. These proteins facilitate adherence to host tissues and enable the bacterium to evade the immune response. 

The expression of *mga* is influenced by the growth phase of the bacteria. During exponential growth, high levels of Mga are maintained, which is crucial for the activation of its target genes. As the bacteria enter the stationary phase, however, the expression of *mga* can be downregulated. Mga activity is closely linked to carbohydrate availability. In the presence of preferred carbohydrates like glucose, the CcpA regulator promotes high-level expression of *mga* [36]. When glucose becomes scarce, however, the expression of *mga* is reduced, which in turn affects the expression of Mga-regulated genes. This regulation is mediated by the phosphotransferase system (PTS), which senses carbohydrate levels and modulates Mga activity accordingly. Mga itself can autoregulate its expression in response to carbohydrate availability, forming feedback loops that help maintain appropriate levels of virulence factor expression in response to changing environmental conditions [37]. 

Mga binds to specific promoter regions of its target genes, facilitating the recruitment of RNA polymerase and the transcriptional machinery necessary for gene expression. Mga binds to promoters as a homo-dimer [38]. Mga binding sites vary in their location relative to the transcription start sites of target genes, and they have been classified into three categories [39]. Initial studies have identified a consensus sequence that Mga recognizes, but the exact binding sites can vary significantly among different promoters [40].

Mga can oligomerize, which enhances its ability to bind to multiple sites within a promoter region. This oligomerization is essential for effective transcriptional activation [41]. Studies have shown that the C-terminal region is necessary for Mga oligomerization [41]. Additionally, Mga activity can be modulated by post-translational modifications, particularly phosphorylation. Phosphomimetic mutations in Mga can disrupt its ability to activate transcription, indicating that non-phosphorylated forms of Mga are more effective in activating target genes [37].

The ability of Mga to activate a diverse array of target genes crucial for virulence, coupled with its regulation by environmental cues and interactions with other regulatory systems, makes it a key player in the pathogenicity of *S. pyogenes* and a potential therapeutic target for combating infections. Inhibition of Mga function could significantly attenuate the virulence of *S. pyogenes*. Studies have consistently shown that *S. pyogenes* strains lacking proper Mga activity exhibit significantly reduced virulence compared to wild-type strains [37,42]. For example, in mouse models of necrotizing fasciitis, Mga knockout mutants were found to be less capable of causing severe disease [42].

**Figure 2:**
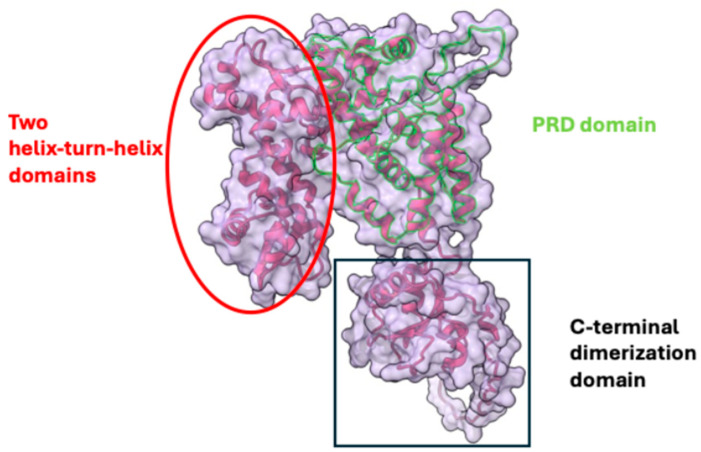
Structure of the Mga monomer. The structure is illustrated based on AlphaFold prediction available in the UniProt Knowledgebase (UniProtKB) database (data label: Q9A0L5) and was generated using Chimera X-1.8. The crystal structures of orthologs in other bacteria such as those in *Enterococcus faecalis* (3SQN in RCSB PDB) and *S. pneumoniae* (5WAY in RCSB PDB) form dimers using the C-terminal dimerization domain, suggesting *S. pyogenes* Mga could form dimers within cells. When the dimer binds DNA using the two helix-turn-helix domains, it would oligomerize to activate transcription. Mga is a regulatory protein whose activity is influenced by the Phosphotransferase system Regulatory Domain (PRD). The PRD plays a crucial role in the phosphorylation process, which involves transferring phosphate groups to specific histidine residues. This phosphorylation affects how Mga functions and its capability to control gene expression linked to metabolism and virulence in *S. pyogenes*.

Potential targets for developing Mga activity-inhibiting agents include the phosphorylation sites and oligomerization sites (Figure 2). A more feasible target would likely be the oligomerization site, as phosphorylating or altering the charge of certain amino acids or regions can be challenging. Chemical agents that bind to the Mga C-terminus and inhibit its oligomerization can effectively abolish the function of Mga, thus serving as anti-virulence agents.

### 3.3. CovRS

Bacterial two-component regulatory systems (TCSs) consist of sensor kinases and response regulators. Sensor kinases detect environmental signals and undergo autophosphorylation, subsequently transferring a phosphate group to response regulators. This phosphorylation event alters gene expression. *S. pyogenes* has been found to possess an average of 13 TCSs [43]. Among these, the CovRS system (also known as CsrRS) plays a crucial role in the pathogenesis of *S. pyogenes*. This system responds to environmental stresses and regulates various virulence factors. 

CovS is an integral membrane protein that functions as a sensor kinase. CovS senses various environmental stimuli such as magnesium ions [44] and a human antimicrobial peptide [45]. These stimuli trigger autophosphorylation of CovS at a specific histidine residue. Once autophosphorylated, CovS transfers the phosphate group to a specific aspartate residue on CovR, the response regulator. This phosphorylation event modulates the activity of CovR, leading to changes in the expression of target virulence genes. CovR regulates multiple virulence factors, both directly and indirectly. CovR represses the expression of the genes of hyaluronic acid capsule operon (*hasABC*), streptolysin S (*sagA*), a protease (*speB*), streptokinase (*ska*), and streptodornase (*sda*) [46,47]. Mutations in the *covS* or *covR* genes can lead to a loss of function or altered function of the regulatory system, resulting in hypervirulent strains of *S. pyogenes* [48]. These strains often exhibit increased expression of virulence factors, enhanced survival in the host, and greater capacity to cause severe disease.

**Figure 3:**
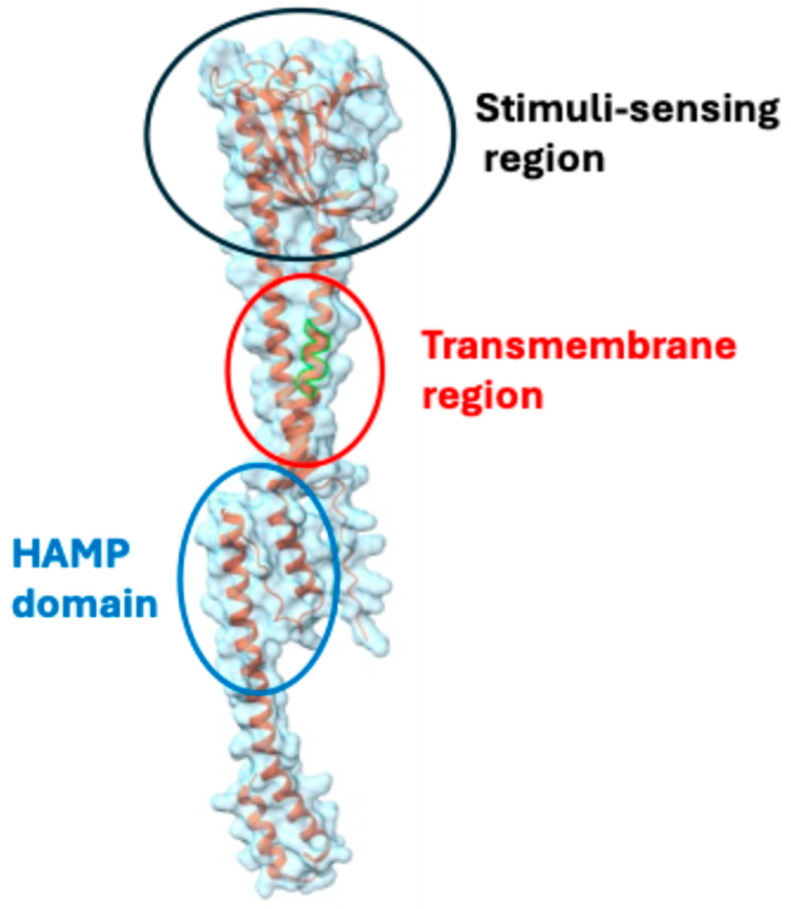
Structure of CovS. This figure illustrates the structure of the CovS monomer, based on AlphaFold prediction (B3VA65 in UniProtKB) and was generated using Chimera X-1.8. CovS is an integral membrane protein that spans the membrane through its transmembrane region. The portion of the protein containing the signal-sensing domain is exposed to the environment, while the HAMP domain is located in the cytosol. The HAMP domain plays a crucial role in signal transduction in various microbial signaling proteins, including histidine kinases [50]. Located between the transmembrane helices and the output domains of signaling proteins, the HAMP domain is a key mediator of signal transduction, connecting input signals to appropriate cellular responses. HAMP domains typically consist of two amphiphilic helices. These helices come together to form a structure known as a dimeric four-helical bundle. Thus, the CovS protein is expected to form dimers in cells.

Since the CovRS system significantly influences the bacterium’s ability to cause disease, it represents a potential therapeutic target for combating infections caused by this pathogen. One potential target site is the environmental stimuli-sensing domain of CovS (Figure 3). If a chemical binds to CovS and increases the phosphorylation activity of CovS, CovS would stimulate the phosphorylation of CovR. The phosphorylated CovR, acting as a repressor [49], would then suppress the expression of the aforementioned virulence genes. However, this sensing domain has not been well studied, so it is necessary to explore the detailed mechanism of how the sensing domain senses stimuli. Another potential target is the phosphorylation relay. However, since human cells have many phosphorylation relay or cascade systems, it might not be easy to develop an inhibitor specific to the phosphorylation relay of CovRS. 

### 3.4. Cyclic di AMP Signaling Pathway

The cyclic di-AMP (c-di-AMP) signaling pathway is vital for bacterial survival and pathogenesis. This molecule regulates various essential cellular processes, enabling bacteria to adapt and thrive in diverse environments. One of its key functions is managing osmotic stress by controlling the uptake and release of osmolytes, such as potassium ions, which maintains osmotic balance and cell viability [51]. Additionally, c-di-AMP is crucial for maintaining cell wall integrity, thus ensuring structural robustness against environmental stressors [52]. It also plays a significant role in bacterial metabolism, particularly under stress conditions, by influencing metabolic pathways through the modulation of enzyme and transporter activities. This modulation helps bacteria optimize energy usage and adapt to nutrient availability [53]. Furthermore, c-di-AMP can be detected by the host’s immune system via the cGAS-STING pathway, triggering an immune response. To evade detection, some bacteria adjust their secreted c-di-AMP levels, which aids in immune evasion and promotes persistent infections [54].

Building on its critical role in general bacterial functions, c-di-AMP is particularly essential in the pathogenesis of *S. pyogenes* by regulating various cellular processes that are crucial for bacterial survival and virulence [55]. Cyclic di-AMP enables *S. pyogenes* to handle environmental stressors such as high salt concentrations, low pH, and reactive oxygen species. *S. pyogenes* lacking the c-di-AMP synthase enzyme, CdaA (aka DacA), show increased vulnerability to these stressors, highlighting vital roles of c-di-AMP in stress management. 

Moreover, c-di-AMP is involved in biofilm formation, which is essential for bacterial persistence and resistance to host immune defenses. Without c-di-AMP, *S. pyogenes* cannot form detectable biofilms, underscoring its importance in this process. Additionally, c-di-AMP is crucial for producing virulence factors, particularly SpeB, a major contributor to the pathogenicity of *S. pyogenes*. Both CdaA and the c-di-AMP phosphodiesterase Pde2 are vital for SpeB production. Mutants lacking one of these proteins exhibit significantly reduced virulence in an animal model [55]. 

Although c-di-AMP is not necessary for growth in nutrient-rich environments, its dysregulation can lead to multiple adverse effects that hinder the disease-causing abilities of *S. pyogenes*. Given its pivotal role in regulating virulence and stress responses, the c-di-AMP signaling pathway is a promising target for developing anti-virulence drugs.

**Figure 4:**
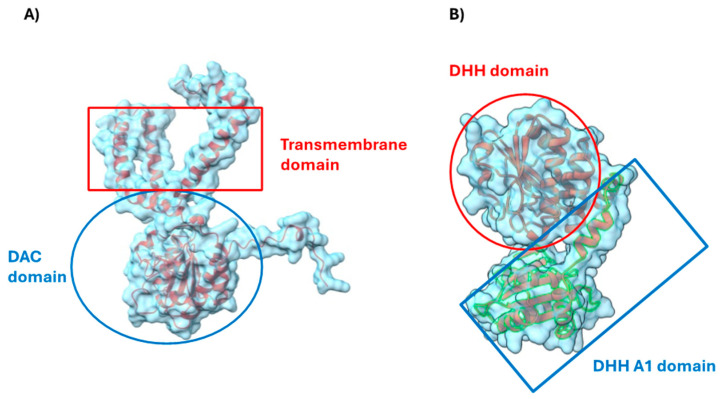
Structure of CdaA (**A**) and Pde2 (**B**). (**A**) This figure illustrates the structure of the CdaA monomer, based on AlphaFold prediction (Q99ZX0U in ProtKB) and was generated using Chimera X-1.8. (**B**) This figure shows the Pde2 monomer structure based on the AlphaFold prediction (Q9A0L5 in UniProtKB).

CdaA, an integral membrane protein, spans the membrane through its transmembrane domain and has a cytosolic Di-Adenylate Cyclase (DAC) domain (Figure 4A). It is uncertain if the minimal extracellular portion contains a signal-sensing domain. Orthologous structures, like those in *Listeria monocytogenes* (6HVL in RCSB PDB) and *S. pneumoniae* (8OFG in RCSB PDB) form dimers via the DAC domain, suggesting potential dimerization in cells. Given that the DAC domain exhibits enzymatic activity and is involved in dimerization, it could serve as a target for developing anti-virulence agents. Pde2 is a cytosolic protein containing DHH and DHHA1 domains (Figure 4B). These domains possess phosphodiesterase activity, making them potential targets for inhibitor development. The ortholog NrnA in *Staphylococcus aureus* (8IU7 in RCSB PDB) forms a tetramer, suggesting *S. pyogenes* Pde2 might also tetramerize in cells.

## 4. Concluding Remarks

The increasing prevalence of antibiotic resistance in *S. pyogenes* necessitates a shift in therapeutic strategies. The exploration of anti-virulence agents represents a promising avenue to combat infections by targeting the regulatory mechanisms that govern virulence factor expression. By focusing on molecular targets such as the RopB, Mga, CovRS, and c-di-AMP signaling systems, we can develop novel treatments that reduce the virulence of *S. pyogenes* without exerting selective pressure for resistance. Although anti-virulence factors hold great promise, they are unlikely to entirely replace antibiotics. Instead, they may serve as valuable adjuncts to antibiotic therapy, particularly in the fight against antibiotic-resistant bacteria. When used in combination with antibiotics, these agents may enhance the efficacy of existing drugs and resensitize resistant bacteria. Therefore, combining anti-virulence strategies with traditional antibiotics represents a more comprehensive approach to managing bacterial infections.

Anti-virulence strategies hold promise in reducing the likelihood of resistance development. Unlike traditional antibiotics, anti-virulence agents do not directly threaten bacterial survival, thereby lowering the evolutionary pressure for resistance. However, the potential for resistance cannot be entirely dismissed. Targeting a master regulator in signal transduction pathways may disrupt homeostatic regulation points, significantly affecting adaptation processes. As a result, this approach could generate selective pressure, potentially leading to resistance through intrinsic mutations [56]. Future research should focus not only on identifying and optimizing anti-virulence compounds but also on monitoring their long-term effectiveness including the potential for resistance development.

## Data Availability

Not applicable.
